# A transcriptional time-course analysis of oral vs. aboral whole-body regeneration in the Sea anemone *Nematostella vectensis*

**DOI:** 10.1186/s12864-016-3027-1

**Published:** 2016-09-07

**Authors:** Amos A. Schaffer, Michael Bazarsky, Karine Levy, Vered Chalifa-Caspi, Uri Gat

**Affiliations:** 1 Department of Cell and Developmental Biology, Life Sciences Institute, Hebrew University of Jerusalem, Edmond Safra Campus, Jerusalem, 91904 Israel; 2 Bioinformatics Core Facility, National Institute for Biotechnology in the Negev, Ben-Gurion University of the Negev, Beer-Sheva, 84105 Israel

**Keywords:** Regeneration, Head vs. tail regeneration, *Nematostella vectensis*, Major axis polarization, Wnt pathway, Metalloproteinase, Homeobox genes, Cilia

## Abstract

**Background:**

The ability of regeneration is essential for the homeostasis of all animals as it allows the repair and renewal of tissues and body parts upon normal turnover or injury. The extent of this ability varies greatly in different animals with the sea anemone *Nematostella vectensis*, a basal cnidarian model animal, displaying remarkable whole-body regeneration competence.

**Results:**

In order to study this process in *Nematostella* we performed an RNA-Seq screen wherein we analyzed and compared the transcriptional response to bisection in the wound-proximal body parts undergoing oral (head) or aboral (tail) regeneration at several time points up to the initial restoration of the basic body shape. The transcriptional profiles of regeneration responsive genes were analyzed so as to define the temporal pattern of differential gene expression associated with the tissue-specific oral and aboral regeneration. The identified genes were characterized according to their GO (gene ontology) assignations revealing groups that were enriched in the regeneration process with particular attention to their affiliation to the major developmental signaling pathways. While some of the genes and gene groups thus analyzed were previously known to be active in regeneration, we have also revealed novel and surprising candidate genes such as cilia-associated genes that likely participate in this important developmental program.

**Conclusions:**

This work highlighted the main groups of genes which showed polarization upon regeneration, notably the proteinases, multiple transcription factors and the *Wnt* pathway genes that were highly represented, all displaying an intricate temporal balance between the two sides. In addition, the evolutionary comparison performed between regeneration in different animal model systems may reveal the basic mechanisms playing a role in this fascinating process.

**Electronic supplementary material:**

The online version of this article (doi:10.1186/s12864-016-3027-1) contains supplementary material, which is available to authorized users.

## Background

### The regeneration phenomenon in animals

How some animals readily manage to reform lost or damaged body parts has always intrigued man and represents a major classical biological riddle. This phenomenon was first subjected to scientific examination by early researchers/naturalists such as Abraham Trembley in the mid-18th century, who conducted elaborate experiments on a cnidarian he termed Hydra [1], and later, at the turn of the 20th century, by Thomas Morgan, who explored many plants and animals including Hydra and the flatworm Planaria [2, 3].

In its broader sense, regeneration results in the compensatory growth of any injury or detraction from the animal body, such as that which occurs during wound healing. However, researchers in the field define this term as a major restoration of body parts in a process that resembles some aspects of embryonic development (reviewed in [4–8]). The ability to restore organs and whole body parts is widespread in animals and also quite common in vertebrates, albeit limited in its scope to body parts like limbs and tail. The underlying mechanism responsible for this extraordinary regeneration ability is thought to be the deployment of pluripotent stem cells (in Planaria known as neoblasts), which can produce all cell types in response to injury or dissections [6, 8–11].

Cnidarians have remarkable regenerative abilities, and Hydra has served as a model organism for this purpose for almost three centuries since Tremblay’s time [12]. Hydra was found to be the most regenerative animal explored to date, because it can regenerate from small parts, and can even form new animals from dissociated cell masses, a phenomenon not observed in other more complex animals (reviewed in [12–15]). This great regeneration potential is also thought to be mediated by stem cells, which give rise to all of the Hydra cell types [8, 16, 17]. Many of the key molecular players involved in the regeneration of Hydra have been identified, and include Wnt3 as a major head inducer, six additional Wnts, and members of the canonical Wnt pathway, including the major effector β-catenin and its DNA binding partner Tcf [18–22]. The Wnt pathway is most likely to be conserved in all of the cnidarian regeneration systems and was shown to also be active in the vertebrate limb [23] and in Planarians [24–29].

#### Polarized regeneration

The open question of how the amputated animals retain their proper polarity while regenerating still represents a mystery. In Hydra, upon mid-gastric bisection, asymmetric molecular events occur, wherein interstitial lineage cells in the head (oral)-facing part of the regenerate, undergo cell death, which leads to Wnt3 secretion and head organizer induction, resulting in subsequent cell proliferation and head formation [30–32]. In the foot (aboral)-facing part of the regenerate, little cell death is observed and thus no head organizer forms, subsequently leading to foot formation. The cell death was found to be induced by the injury-driven asymmetrical activation of the ERK/MAPK pathway, which was detected only in head-facing wounds [33, 34]. However, in Hydra undergoing regeneration after decapitation, there is no asymmetrical cell death and no involvement of interstitial stem cells, which are not present in this region, but rather, the epithelial cells respond to injury signals and release *Wnt3* [34].

In Planaria, *Wnt1*, which is necessary for executing the posterior (tail) regeneration program, does not exhibit polarized expression upon amputation and becomes enriched at the posterior-facing wounds only at later stages of the process [28]. The gene that does exhibit asymmetrical expression upon dissection and appears only at anterior wounds is *Notum*, which inhibits the activity of Wnt factors and also serves as a target gene of the pathway [29]. Recently, Wurtzel and colleagues reported that *Notum* was the first gene to show polarized expression in the time course of Planarian regeneration [35]. The execution of the head-to-tail polarity in Planaria is thus thought to be controlled by gradients of several Wnt factors that are expressed at different times after initiation of regeneration, and in different anterior-posterior (AP) domains, with feedback inhibition from *Notum* and other anterior inhibitors such as sFRPs [26, 28, 29]. The Wnt pathway is key to the polarized regeneration program because it establishes the A-P axis upon embryonic development in most bilaterians and in the basal metazoans at large [26]. Several other developmental pathways (e.g., Hedgehog, FGF, BMP) are also instrumental for polarized regeneration as well as many transcriptional regulators, which are asymmetrically expressed and most likely play crucial roles in this complex developmental feat [36–39].

#### Regeneration in *Nematostella*

The starlet sea anemone, *Nematostella vectensis* (Nv), belongs to the basal cnidarian class Anthozoa and is now a well-established model organism, owing to its ease of culturing under simple laboratory conditions [40–42]. It can reproduce both sexually, undergoing embryonic and planula larva stages, and asexually [40, 42, 43]. The availability of the *Nematostella* genome revealed its unexpected complexity in terms of its rich genetic repertoire [44] and molecular genetic studies demonstrated that the main axis of the animal (aboral to oral) bears a resemblance to the bilateral AP axis as it exhibits some differential expression of *Hox* genes and contains anterior markers in the aboral region and typical posterior markers (like Wnts) in the oral part [42, 45–51].

*Nematostella* undergoes regeneration after transverse dissection; thus, it generates two complete and viable animals and also sometimes responds to injuries by forming extra heads or physa [43]. Like some of the sea anemones, the mode of its asexual reproduction is by transverse fission called physal pinching and occasionally it also performs polarity reversal; both of these events resemble regeneration. The main morphological stages that can be observed in *Nematostella* undergoing head regeneration from aboral (physa) fragments [52] and most recently after sub-pharynx amputation [42, 53] have been described and specific morphological stages were assigned.

The cell dynamics in *Nematostella* is expected to be very different from that of Hydra, since dividing cells in sea anemones, e.g. *Aiptasia*, are not limited to the main body column as is in Hydra, but instead are distributed in an oral-aboral gradient with the highest level of proliferating cells present in the oral/tentacle region [54]. In *Nematostella* undergoing oral regeneration, proliferation of cells started about a day after dissection [55]. Inhibition of cell division aborted the regeneration, demonstrating that unlike Hydra, which exhibits “morphallactic” regeneration, the mode of regeneration in *Nematostella* is “epimorphic-like” and relies on cell proliferation. Amiel et al. reported that cell proliferation started at 12 h after head amputation, was followed by later periods at 24–48 h, and that proliferation is necessary for the later stages of regeneration but not for the initial wound healing stage [53]).

Regarding molecular studies, like in the Hydra, the Wnt pathway has been shown to be involved in oral regeneration, as demonstrated by treating regenerates with alsterpaullone, a Wnt pathway activator. This led to ectopic oral structures growing from polyps undergoing aboral regeneration and from wounds in its torso region [56]. Recently, a genome-wide microarray-based transcriptional profiling screen was conducted to obtain the expression patterns of genes during the early wound healing process of regeneration. The healing process was observed during a few hours after puncture wounds were performed in the aboral region of juvenile polyps [57]. A group of wound-healing response genes, some of which, like metalloproteinases and several transcription and growth factors, were found to be induced also in many other species [37, 58], whereas other genes were specific for *Nematostella*. When pharmacological inhibitors were used, the ERK/MAPK pathway was shown to be essential for wound healing and regeneration in *Nemtostella*, similar to the previously reported Hydra and other wound-healing systems such as in *Drosophila* imaginal discs [32, 58].

To date, the oral vs. aboral regeneration response in *Nematostella*, which is currently the most basal metazoan model animal for the study of regeneration, has not been addressed. While Hydra and Planaria exhibit the highest regeneration capacities in terms of the extent of body rebuilding ability [5], likely due to specialized stem cells [6, 8], *Nematostella* may present the most primordial regeneration prototype and is therefore important for understanding the basis of this phenomenon.

Our goal in this study was to obtain insights into the transcriptional regulation programs of oral/aboral regeneration and compare the molecular time-course patterns of the two sides. We therefore used an RNA-Seq screen covering the 72 h of the regeneration process during which wound healing takes place and the animals complete the morphogenesis/patterning stage to assume their overall body shape. This screen was verified and extended by a comprehensive qPCR study of gene expression in the wound-proximal regions. We report the general trends of gene expression along this process and characterize the genes and gene pathways which responded differentially in oral vs. aboral regeneration. We elaborate on the kinetics of genes and gene pathways with polarized expression that are known from previous studies in *Nematostella* and other regeneration models, and reveal newly discovered genes that are likely to be important for the process. A comparison of gene expression patterns is made with the well-studied Planarian anterior-posterior regeneration paradigm, which unmasks evolutionary commonalities and differences in this major re-development event.

## Results and discussion

### General trends of the oral and aboral regeneration transcriptional programs in *Nematostella*

**Fig. 1:**
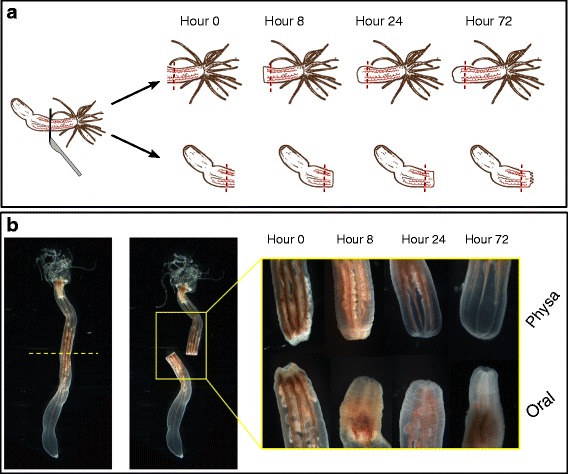
Experimental design of a time-course analysis of regeneration in *Nematostella vectensis*. **a**. A schematic depiction of the experimental procedure. Aboral “tail or physa” regeneration appears in the *top row* and oral “head” regeneration appears in the *bottom row*. The dissection planes are denoted by *dashed red lines* and the wound proximal parts were used for RNA extraction and the RNA-Seq screen. **b**. Photographs of regeneration process corresponding to the time course of the experiment. *Left panels* show a polyp before and after bisection at a plane marked by the *yellow dashed line*. The *right panel* shows pictures of the oral and physal-regenerating parts of the bisected fragments along the time course

In order to study the transcriptional basis of oral versus aboral regeneration we performed a transcriptional profile screen in which we examined the time course of polarized regeneration. To this end, we dissected adult polyps (ca 3 months old) into two parts in their mid-body, which corresponds to mid-gastric dissection in the Hydra. The wound-proximal “blastema”–like regions from 20 oral (‘head’) or aboral (‘tail’ or physa) fragments were taken immediately upon amputation (hour 0) and at three later time points (8, 24, and 72 h post dissection) (see Fig. 1a). These time points represent early regeneration at the wound-healing stage (up to ~8 h), the active growth and cell proliferation stage (24 h), and the later stage of remodeling and morphogenesis at 72 h [42, 52, 53, 55, 57]. The morphological changes in the appearance of the bisected polyps were followed. We observed that at 8 h wound closure was complete due to wound healing processes, as reported by DuBuc et al. [57] for puncture and bisection assays performed on very young polyps [57] and by Amiel et al. [53] for adult and young polyps. At this time we did not find any visible signs of new tissue formation and the wounded mesenteries are seen to be in close proximity to the site of the cut, while at the first hours they seem to recede from the wound plane (Fig. 1b). At 24 h we observed that the mesenteries seemed to be attached to the center of the cut area in both the oral and aboral fragments as reported before for oral regeneration [53, 57]. At 72 h tentacle buds can begin to be detected in the regenerating oral side, and in the regenerating physa the mesenteries appeared disconnected from the previously wounded area and the physal end is more rounded and clear. We chose 72 h as our last time point since the general re-establishment of the body plan after dissection and the major morphogenesis processes seem to have been determined by this time. While the regenerating polyps still grow extensively for several more days until their body parts assume normal proportions, and several more weeks for returning to their original size, this is secondary to the reorganization of the body plan.

We selected those regions close to the wound sites in order to focus on the parts that undergo the most conspicuous changes in their oral-aboral characteristics and in which we expected to observe a high level of response. RNA was extracted from the regenerating oral and physal sides. For each time series of regeneration two independent pools consisting of 20 animals each were created for each of the four time points (Fig. 1a); thus, we prepared 16 Illumina Hi-seq libraries. Sequencing these libraries returned an average of ~24.5 M 50 bp single end reads per sample with a standard deviation (SD) of ~9.2 M reads. An average of 73 % of the reads per sample (SD 3 %) uniquely mapped to the predicted genes in the draft *Nematostella* genome [44]. The number of expressed genes that were detected through read alignment was ~23000, whereas the total number of predicted *Nematostella* genes is ~24000.

To identify genes with time-dependent expression, a multi-factorial design analysis was carried out using generalized linear models (GLM) fitting and inference (see Methods). In this analysis we tested for the time effect on gene expression, taking into account that gene expression may also be affected by the regeneration-side. A total of 4205 genes, which corresponds to ~17 % of all *Nematostella* genes, displayed a significant time dependent change (BH adjusted *p*-value < 0.05). In Additional file 1: Table S1 these differentially expressed, time-course responsive genes (“DE genes”) are listed with their normalized number of reads together with their annotations obtained from UniProt (when available) or with the use of the Blast2GO tool. This table was the basis for the different expression patterns analyses that we present in this study.

**Fig. 2:**
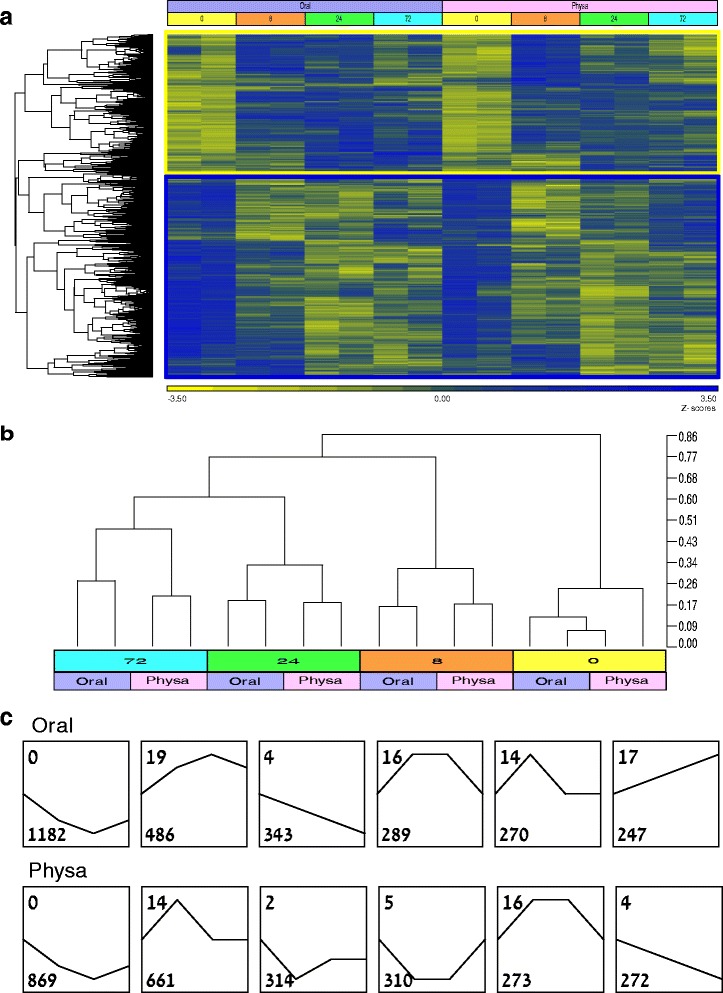
Clustering analyses of the oral and physal time course. **a**. Gene-wise hierarchical clustering of the differentially expressed (DE) genes. The *yellow rectangle* highlights genes being largely upregulated during regeneration. The *blue rectangle* highlights genes largely being downregulated during regeneration. The color scale indicates z-scored expression values **b**. A dendrogram of sample-wise hierarchical clustering of the samples from the different regeneration-sides and time points. **c**. The six largest clusters of gene expression in oral and physal regeneration. Clusters are ordered by the number of genes in each cluster which is indicated in the *bottom left* corner. The number in the *top left* corner indicates the STEM cluster number. The full list of clusters can be found in Additional file 2: Figure S1 and a list of the genes and which cluster they belong to can be found in Additional file 3: Table S2

We first examined the global nature of the gene response pattern in the oral and aboral regenerating parts. A hierarchical clustering analysis of all the DE genes indicated that more genes (about 60 %) were downregulated following dissection compared to the upregulated genes (Fig. 2a). Within the two groups many blocks of variation in one or more time point were evident, indicating temporal-, and tissue-specific differential gene expression. Similarly, sample-wise hierarchical clustering according to the temporal and spatial variables (Fig. 2b) indicates that gene expression patterns of the two tissues were largely shared at each time point, and that the different time points exhibited more distinct expression patterns from each other. As expected, the expression profiles of the two cut ends at time 0 were the most similar to each other and differed from all the ensuing regeneration time points. It was also evident that the 24 and 72 h expression profiles are more similar to each other than they are to 8 h (Fig. 2b), which is in line with previous studies defining the first 6–8 h as a distinct wound-healing stage [53, 57]. The similarity in the overall expression patterns of the two regions of regeneration, at each individual time point, suggests that a large part of the regeneration response is of a general nature which may govern general wound healing and tissue growth at large, and that side-specific differential expression patterns may be more limited. Interestingly though, the oral and physal 72 h samples were more diverse than in the other time points as reflected in their branch lengths (Fig. 2b), which may indicate that at 72 h there is an enriched expression of genes responsible for oral or aboral specific tissue traits compared to a more general earlier wound healing and initial regenerative response.

In a study performed in Planaria, in which the transcriptional profiles of head and tail segments following bisection were compared by RNA-Seq analysis [38], the two parts were highly diverse at the early hours and converged to a similar pattern at the later time points. In this screen, similar to our screen there were also more downregulated than upregulated differential transcripts along most of the time course. Unlike our protocol, however, the Planaria study analyzed the entire regenerating fragments and not the wound-proximal regions as in our study, which explains the arrival to a similar state of gene expression towards the culmination of the regeneration process.

In order to identify and characterize the distinct expression profiles presented by each of the regeneration parts, we used the Short Time-series Expression Miner (STEM) software. This software is specifically tailored for high-throughput time series gene expression datasets having small number of time points with few replicates [59]. Using STEM clustering algorithm, we grouped the DE genes to clusters representing the major expression profiles in the oral and physal sides (Fig. 2c). Similar to the hierarchical clustering, the STEM clustering showed that the largest and most prominent cluster was of consistently downregulated genes. Most of the clusters in one of the sides have similar counterparts in the other side and indeed there are many shared genes in these clusters, however, the gene numbers in the clusters vary between the sides (Fig. 2c) due to side specific gene trends. The complete profile of all oral and physa clusters and their genes is presented in Additional file 2: Figure S1 and Additional file 3: Table S2.

**Fig. 3:**
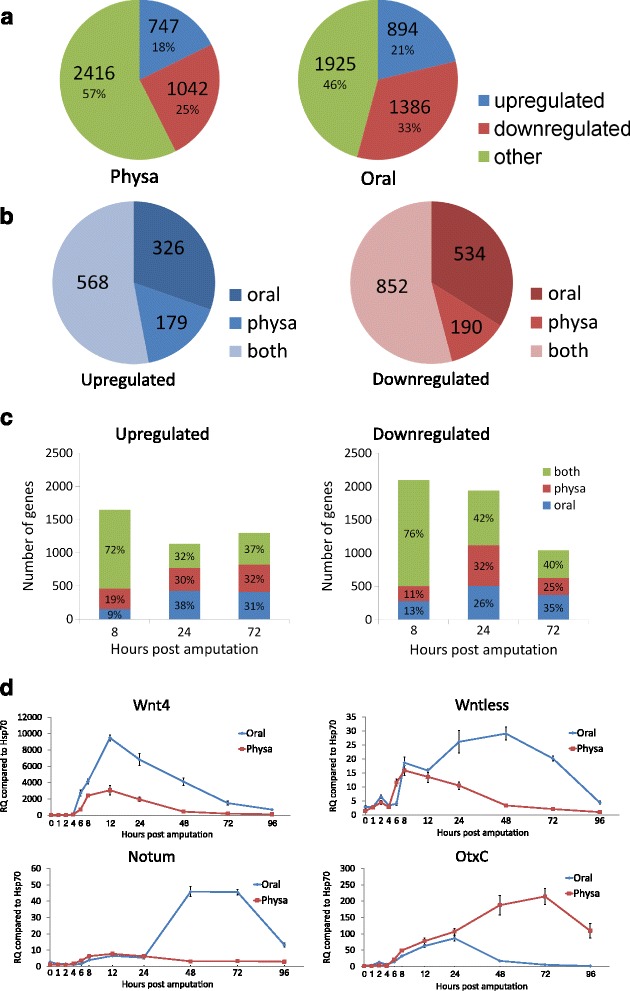
A general analysis and comparison of side and time course expression trends. **a**. Distribution of genes in either the oral or physal regenerating sides according to their time course response pattern: always upregulated, always downregulated or other, having a more complex trend. Genes were considered always up or downregulated if all time points of regeneration were at least 1.6 fold higher or lower than hour 0, respectively. **b**. Distribution of genes being either always upregulated or downregulated according to their oral or physal response in regeneration. **c**. The number of oral or physal genes being up or downregulated at each time point along regeneration. Genes were considered up or downregulated at each time point if their expression was at least 1.6 fold different compared to their expression level in the previous time point **d**. Examples of qPCR results of highly DE genes that show a similar expression pattern in both sides during early regeneration and a prominent divergence during later regeneration

Further analysis of the general time course trends revealed that about half of the DE genes could be defined as being always up or down-regulated throughout the sampled time points of regeneration, while the remaining genes displayed more complex expression patterns, with one or more changes in the direction of expression along the time course (Fig. 3a). We characterized a gene as being always upregulated if all time points of regeneration showed an expression level higher than that of hour 0, and always downregulated if expression in all time points were lower than in hour 0. When examining the expression modes in the overall always up and downregulated genes, the largest group consisted of genes that behaved similarly in both sides (~50 %), while the rest were either oral or physal specific (Fig. 3b). Of the genes that are regulated in one side, the oral side contained more of these and this was particularly evident among the downregulated genes. A list of the genes and their trend of expression can be found in Additional file 3: Table S2.

For the comparison between time points we examined whether expression was being up or downregulated compared to the previous hour of regeneration. At the early regeneration stage (8 h), the majority of the DE genes (~75 %) displayed the same expression trend (up or down) in both the oral and physal sides (Fig. 3c). During the later stages of regeneration, a minority of genes exhibited such a parallel expression pattern and the majority showed different trends of expression in the two sides. Thus, the early response was more similar between the sides and also comprised a larger number of responsive genes, while at the later stages we observed a larger proportion of side specific genes. There was a cumulative increase in side specific upregulated genes up to 72 h whereas the number of side-specific downregulated genes decreased dramatically between 24 and 72 h, suggesting that gene upregulation characterizes tissue specific regeneration (Fig. 3c).

Similarly when we followed the time course of individual genes which displayed prominent differences in expression between the sides, we often detected similar expression at both sides in early regeneration followed by a divergence in the expression pattern. This fits the general trend we observed in our global analysis of diversification with time. An example of such an expression pattern, verified by qPCR at a higher time resolution, can be seen for the gene *Wntless* (Fig. 3d), which is an important regulator of Wnt factors and was implicated in polarized regeneration in Planaria [60]. This profile is typical for many of the polarized genes, both oral as shown for *Wnt4* and *Notum*, and aboral as shown for *OtxC* (Fig. 3d). Thus, it seems that often the initial gene response in the two sides is similar and with time the appropriate pole-specific gene expression pattern is established. This suggests an interesting strategic mechanism of “repair missing parts first and implement polarization later”. However, the question remains whether this early response is due to a dual functionality of these genes with an early non side-specific wounding/regeneration function and a later side specific role, or whether the similar expression pattern in the immediate response is due to the animal creating a reserve of available strategic transcripts for use in the regeneration response, which will be utilized eventually only in the appropriate side with the establishment of polarity. This scenario greatly differs from the results reported for a head vs. tail RNA-Seq regeneration screen in Planaria, where the transcriptional profiles of many genes were very different at the start and later on converged to a similar pattern [38]. However, this difference is likely due to the experimental strategy used in the Planaria study of examining the entire fragments rather than the proximal regenerating regions. Yet, the initial similarity in expression between the sides in our screen does not mean that some key regulators are not differentially expressed from the start in *Nematostella* regeneration and indeed, we detected such early asymmetric genes, which may participate in determining polarity already at an early stage as will be reported ahead.

### Characterization of side specific gene responses in regeneration

**Fig. 4:**
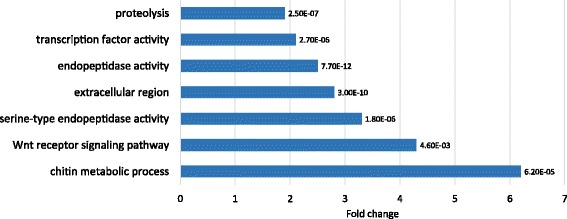
Major Gene Ontology (GO) terms with highly differential oral to aboral expression. Fold change of the most prominent GO terms that are enriched in genes with low concordance between the sides is shown. The numbers next to bars represent Benjamini-Hochberg (BH) adjusted *p*-values for the enrichment of the GO term

Next, we embarked on our main aim of elucidating the molecular basis of the oral vs. aboral (physal) regeneration programs, which is paramount to understanding the nature of the polarized regeneration logic. In order to characterize differentially expressed genes as tissue-specific we tested several methods of correlation analysis of the time course results. We found that the concordance method for correlation calculation (CCC) best revealed differences in both geometry and levels of gene expression. According to this analysis, genes with a low CCC are less similar and more side-specific in their expression pattern. A list of all time course responsive genes in the screen according to their CCC score, together with their oral or aboral nature and their response ratios in the time span of their largest change, is found in Additional file 3: Table S2. For a systematic identification of functional gene groups we considered genes to have a low correlation if they had a CCC of 0.6 or lower and analyzed them for enriched GO terms (as described in Methods). 1804 genes out of the 4205 time responsive genes passed this cutoff. In Table 1 the top most differentially expressed genes out of this list with the above data is shown. It can be clearly observed that the table contains a striking abundance of two types of gene groups: transcription factors and Wnt pathway members. The major GO groups that were detected in the list and their enrichment fold are depicted in Fig. 4 and a full list of the major GO groups with low CCC can be found in Additional file 4: Table S3. Some of the GO categories are redundant while some are a combination of other groups, for instance the large “extracellular region or matrix” groups are composed of the protease/peptidase groups, the Wnt pathway group and the chitin related group. The main functional groups that we found to be enriched among the low concordance genes are described:

**Table 1:** Differentially expressed oral vs. aboral genes

ID	Annotation	Adjusted *p*-value	Concordance	Higher activity in	Hour of strongest change	In side	Level of log highest change
v1g28237	Transcription factor sp5	7.14E-07	−0.17115	Oral	Hour 24	Oral	2.9849
v1g83478	Lactose-binding lectin l-2- partial	1.66E-07	−0.12819	physa	Hour 72	Physa	2.00183
v1g130873	Homeobox protein six3 (Nv-Six3/6)	1.58E-06	−0.12477	physa	Hour 24	Physa	2.0107
v1g213735	OtxC	8.12E-11	−0.07607	physa	Hour 8	Physa	4.721525
v1g234699	Twist-related protein 2-like	2.02E-08	−0.07025	Oral	Hour 24	Oral	7.370836
v1g128302	MoxB	8.11E-07	−0.06785	Oral	Hour 24	Oral	5.729278
v1g240144	Predicted protein	1.52E-08	−0.03445	physa	Hour 8	Oral	4.599914
v1g187332	Forkhead box protein b1	4.09E-07	−0.02903	Oral	Hour 8	Oral	2.974586
v1g236657	Protein	1.72E-06	−0.01588	physa	Hour 24	Physa	2.600729
v1g19039	Transcription factor 24-like	2.55E-08	−0.00569	Oral	Hour 72	Oral	3.74859
v1g128296	Homeobox protein mox-2-like	4.21E-11	−0.00251	Oral	Hour 72	Oral	6.851678
v1g143839	dbh-like 1	1.10E-08	0.00088	physa	Hour 24	Physa	5.102618
v1g128275	Homeobox protein mox-1-like	2.02E-07	0.00604	Oral	Hour 24	Oral	4.413361
v1g158342	**Protein Wnt (Nv-Wnt1)**	1.73E-13	0.01381	Oral	Hour 72	Oral	5.89264
v1g209762	**protein notum homolog**	9.33E-08	0.01413	Oral	Hour 8	Oral	2.961176
v1g91822	**Protein Wnt (Nv-WntA)**	7.02E-06	0.05128	Oral	Hour 24	Oral	4.354627
v1g204932	Tryptophanase	2.68E-13	0.05226	Oral	Hour 24	Oral	4.082363
v1g209293	Protein	8.58E-08	0.06309	Oral	Hour 72	Oral	5.050021
v1g128289	Homeobox protein mox-1	1.36E-14	0.07061	Oral	Hour 24	Oral	6.652978
v1g90018	Serine threonine-protein kinase nek10	2.42E-06	0.09293	Oral	Hour 24	Oral	1.887118
v1g99612	Transcription factor sp9	4.12E-06	0.10237	Oral	Hour 24	Oral	2.906741
v1g82259	Camp-dependent protein kinase regulatory chain	2.54E-07	0.10922	physa	Hour 24	Physa	4.219632
v1g79707	Diencephalon mesencephalon homeobox protein 1(Nv-DMBXa)	4.70E-10	0.11036	Oral	Hour 72	Oral	4.284746
v1g235967	Transcription factor sox-14 (Nv-Sox1)	3.13E-06	0.11449	Oral	Hour 24	Oral	2.215064
v1g242584	**Protein Wnt (Nv-Wnt2)**	6.25E-14	0.12361	Oral	Hour 72	Oral	3.400295
v1g205060	Tetratricopeptide repeat protein 34-like	4.30E-09	0.12377	physa	Hour 8	Physa	2.469459
v1g211802	Glutamine-rich protein 2	1.75E-06	0.12696	physa	Hour 72	Physa	4.404417
v1g101383	A disintegrin and metalloproteinase with thrombospondin motifs 17	6.16E-08	0.13962	Oral	Hour 8	Oral	4.0445
v1g33652	NA	0	0.1429	Oral	Hour 24	Oral	4.929053
v1g25772	NA	4.83E-11	0.1469	Oral	Hour 24	Oral	5.117176
v1g194914	**Protein Wnt (Nv-Wnt4)**	0	0.15175	Oral	Hour 24	Oral	7.726335
v1g121336	**Protein apcdd1**	1.01E-06	0.15885	physa	Hour 8	Physa	2.812523
v1g228590	Cathepsin l-like	0	0.16234	Oral	Hour 24	Oral	5.303226
v1g247951	Chitin deacetylase 9 precursor	2.24E-12	0.17734	Oral	Hour 72	Oral	5.023155
v1g100430	**Protein wntless homolog isoform 1**	0	0.18089	Oral	Hour 72	Oral	3.727024
v1g196726	Integrin alpha-8	1.64E-11	0.19342	Oral	Hour 24	Oral	2.564167

Some of the most prominently DE genes of the screen are presented sorted by their concordance correlation coefficient (CCC). The column ‘higher activity’ notes the side of regeneration in which there was a higher level of expression in most of the sampled hours. The column ‘hour of strongest change’ indicates the hour with the largest change in expression levels between hours of regeneration and notes the side of the strongest change (log_2_) in expression level. Wnt family genes are marked in bold. Homeobox genes are underlined

#### Peptidases and proteases

These gene families are known to take part in tissue remodeling in many regeneration models ([57] and references therein). These are necessary for the wound healing and injury repair program [61] taking place in both sides. This entails breakdown of the mesoglea, the basal lamina, at the amputation site, and its reconstruction after wound closure, as well as release of growth factors for the following reconstruction phase. Massive protease involvement in the early stages was observed in the previous study on wound healing in *Nematostella* [57], and indeed our screen shows a wealth of many types of such gene groups. Interestingly, but as may be expected, the “proteases”, “peptidases” and “extracellular regions” categories were enriched in the differentially regulated genes groups. This can indicate the widespread differential tissue and extracellular matrix (ECM) sculpting programs and growth factor shedding in the two sides (Fig. 4). Predominantly, the group of zinc metalloproteinases (MPs) of the *ADAMTS* (A Disintegrin And Metalloproteinase with Thrombospondin Motifs) type [62, 63] were differentially expressed in the two sides, with the majority highly expressed at the oral side at some or all time points, while several were expressed higher in the aboral side, as shown in Additional file 5: Table S4 and Additional file 6: Figure S3. Some other MPs showed polarized expression as well, including several members of the Astacin group, that were reported to be essential for both head and foot regeneration in the Hydra (reviewed in: [64]), and also the membrane type ADAM10, which is a sheddase of cell surface proteins such as growth factors. *Nematostella matrix metalloproteinase* (*MMP*) *17* was detected to be oral responsive. Its Hydra ortholog *HMMP* was also reported to be expressed in development and regeneration [64], and the Planarian ortholog was found to be enriched in the anterior regeneration program of the worm [38]. Regarding MP inhibitors, a *tissue inhibitor of MP (TIMP*) gene was reported before in *Nematostella* wound repair [57], to be expressed early in both the oral and aboral sides after amputation, and in our screen displayed an early higher transient aboral expression followed by an oral only expression at later times, suggesting a complex regulation of tissue consistency along the regeneration process. An additional inhibitor with an exclusive early oral expression pattern was also identified (Additional file 6: Figure S3). This complex and dynamic expression pattern of several proteinase types that are at interplay with their inhibitors is likely to be instrumental for the formation of oral and physal specific features.

The noticeable abundance of multiple *ADAMTS* species is an interesting avenue for further research in order to identify the substrates for these enzymes, which will allow a better understanding of their functions. Some of the orthologs we have detected which include multiple *ADAMTS6-like* but also *16* and *17* are orphan, while *ADAMTS9* is a proteoglycanase, *ADAMTS2* and 3 are procollagen peptidases and *7* and *12*, are cartilage oligomeric matrix protein (COMP) proteases [57], with *COMP* also appearing as a down regulated gene in the screen. It will be necessary to verify the identity of the substrates and importantly – explore the patterns of *ADAMTS* expression and activities during the regeneration course. Many genes with a high correlation score were also found to be associated with the “proteases” and “peptidases” GO terms although these terms were not significantly enriched as for the side specific genes of these categories (data not shown). This is to be expected since there is a large general remodeling response of the tissues in both sides as was described above.

#### Transcription factors

The “DNA binding” and “transcription factor” groups were found to be prominent amongst the side specific genes in our screen both in terms of gene numbers and fold enrichment (Fig. 4). In Table 2, the top differentially expressed transcription factors (TFs) were marked according to their type and differential expression pattern in the oral or aboral sides and the full list is shown in Additional file 7: Table S5. Of these factors the homeobox genes were the most dominant group with multiple representatives (about 1 out of every 3 DE TFs), many of which displayed higher induction of expression. This may be expected of developmental “toolkit genes” that are known to be involved in developmental patterning and in evolutionary changes of the developmental process [65]. There are three Hox genes within the homeobox genes. However, the subgroups with the most members (4 each) are *NK-homeobox* type genes (4), which are considered to be the most ancient type of homeobox genes [66] and the *Mox* genes, which are the most highly induced TFs in our screen. Interestingly, the *Mox* family is represented by twice the number of genes in *Nematostella* as compared to human [50]. We can speculate that there may be a connection between this high copy number and strong induction of the *Mox* genes to a specific function in regeneration, which will be explored in future studies. The screen also included three aboral expressed *Six* genes including *Six3/6*, which is a master aboral pole regulator in *Nematostella* development [51], a key anterior development gene in bilaterians in general, and is necessary for forebrain formation in vertebrates [67].

**Table 2:** Differentially expressed homeobox genes and other transcription factors

Gene id	Annotation	Adjusted *p*-value	CCC	Overall side trend	Tmax relative to T = 0^a^ (Hours)	Side of max change/Fold change(Log2)	Fold change (Log2)
Homeobox genes							
v1g184843	Retinal homeobox protein rx-like	0.000375	−0.43	Oral	Hour 72	Physa	2.95
v1g127828	Homeobox protein nkx-(Nk2.2-like)	0.000189	−0.24	physa	Hour 72	Physa	2.43
v1g208035	NK-4 homeobox protein (NvNK-4)	0.000174	−0.14	physa	Hour 8	Physa	1.74
v1g130873	Homeobox protein six3 (NvSix3/6)	1.58E-06	−0.12	physa	Hour 72	Physa	2.01
v1g213735	NvOtxC	8.12E-11	−0.08	physa	Hour 72	Physa	4.72
v1g128302	MoxB (NvMox2)	8.11E-07	−0.07	Oral	Hour 72	Oral	5.73
v1g128296	Homeobox protein mox-2-like (NvMoxD)	4.21E-11	0	Oral	Hour 72	Oral	6.85
v1g128275	Homeobox protein mox-1-like (NvMoxA)	2.02E-07	0.01	Oral	Hour 72	Oral	4.41
v1g128289	Homeobox protein mox-1 (NvMoxC)	1.36E-14	0.07	Oral	Hour 24	Oral	6.65
v1g79707	Diencephalon mesencephalon homeobox protein 1	4.7E-10	0.11	Oral	Hour 24	Oral	4.28
Other transcription factors							
v1g28237	Transcription factor sp5	7.14E-07	−0.17	Oral	Hour 24	Oral	2.98
v1g200081	t-box transcription factor tbx15	1.64E-05	−0.05	Oral	Hour 72	Oral	4.03
v1g187332	Forkhead box protein b1 (NvFoxB)	4.09E-07	−0.03	Oral	Hour 72	Oral	2.97
v1g165603	Forkhead domain protein D1 (NvFoxD1)	0.02904	−0.03	physa	Hour 72	Oral	3.54
v1g67043	Forkhead box protein l2	0.0325	−0.01	physa	Hour 8	Physa	1.86
v1g88753	t-box transcription factor tbx18	0.03405	0	Oral	Hour 72	Oral	3.99
v1g235967	Transcription factor sox-14 (NvSox1)	3.13E-06	0.11	Oral	Hour 72	Oral	2.22
v1g211452	ets translocation variant 5 isoform 1	0.03352	0.2	Oral	Hour 24	Oral	1.94
v1g161959	Hypoxia-inducible factor 1-alpha isoform 2	0.04308	0.2	physa	Hour 72	Physa	1.19
v1g101900	Transcription factor sp5	3.11E-05	0.2	physa	Hour 72	Physa	2.09

Genes with low CCC values were sorted by their adjusted time-effect *p*-value. The column “overall side trend” indicates the side with an overall higher level of expression during regeneration. The next columns show the hour of highest change in expression level as compared to hour 0 (Tmax relative to T = 0), the side in which it is observed (Side of max change), and the level of change in expression (Fold change (Log2)). The other columns contain the hour of overall largest change during regeneration (Time interval of max change) and the side (Side of Max change) and level (Fold change (Log2)) of this change

^a^Hour of maximal change relative to time 0

Other types of TF genes we detected include several factors of the following types: *Forkhead* (7 members), *Sox HMG box domain* (7), *SP zinc-fingers containing* (4), *T-box* (4), *Ets domain* (3), *Krueppel-like* (2) and multiple TFs with one copy such as the bHLH factor *Twist-related2,* the *Runt-domain Runx*, *p63* and *HIF-1*.

Most of the differentially expressed homeobox genes and other TFs are more highly induced in the oral side (67 and 61 % respectively), which can reflect the higher complexity of the structures formed at the oral side. When comparing oral side expression patterns to those of Hydra head regeneration, we can observe similar expression pattern along the process, such as the orthopedia-like (*otp*) and *Sox14* genes which are late expressed oral genes both in Hydra as well as in this screen. On the other hand, some factors show a strikingly different pattern between these two cnidarian groups, such as the paired-type homeobox repressor *Dmbx* gene, which is downregulated in regenerating Hydra heads [68] but upregulated in *Nematostella* oral regeneration, and to a lesser degree also in the aboral part. It is more difficult to compare Hydra and *Nematostella* aboral regeneration since there are few reports regarding aboral gene responses in Hydra regeneration. The comparison of gene expression accompanying the regeneration process of these two systems would be interesting, as their aboral parts differ in shape and function; Hydra have an attachment disc whereas the physa of *Nematostella* is simplified and adapted to burrowing.

#### The Wnt pathway

This major developmental signaling cascade was found to represent a most prominent group of genes that was notably enriched in our screen and for which the expression pattern of many of its members was highly polarized. This pathway is known to be essential for establishing A-P polarity in the developmental stages of many diverse animals [26]. It is also known to play a key role in animal regeneration and specifically in cnidarians [13, 19, 20, 22, 57, 69]. An examination of the expression patterns of these genes along the time course of regeneration verified the expected oral predominance in a large majority of them.

**Fig. 5:**
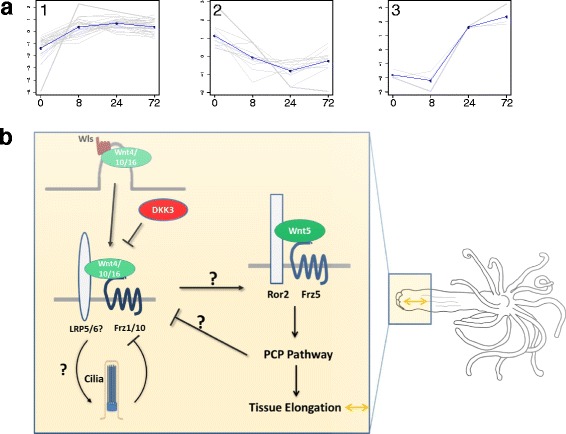
Analysis of the Wnt pathway and other developmental signaling groups. **a**. Clusters of expression of Wnt pathway genes of the GO group in the oral regeneration response. Three clusters were discerned including genes of the following expression patterns (with representative genes): 1. early upregulated (Nv-*Wnt11*, Nv-*Wnt16*, *fzd-10* precursor). 2. downregulated and then upregulated (Nv-*Wnt6* *,10*). 3.late upregulated (Nv-*Wnt1*, Nv-*WntA*). Each gene is plotted (*gray*) in addition to the mean expression profile for that cluster (*blue*). A full list of the genes and their cluster can be found in Additional file 8: Table S6. **b**. A proposed hypothetical schematic of the canonical and PCP Wnt pathways interaction in aboral regeneration. The question marks depict signaling interactions which are less established and are thus more assumptive. The bent arrows represent a possible negative feedback loop between cilia and the early canonical Wnt complex that is highly speculative at this stage

Three distinct clusters of Wnt pathway genes could be identified based on clustering of their oral regeneration expression patterns (Fig. 5a). The largest cluster had an expression pattern in which its genes were upregulated at 8 h and therein either remained upregulated thereafter or showed a decrease in expression but always above their initial time 0 levels. We termed this cluster “early upregulated” (Fig. 5a1) and it contained *Wnts4*, *11* and *16*, *Frizzled1* and *10*, intracellular Wnt pathway members like *Tcf/Lef-1*, *Axin* and *naked-cuticle homolog1*(*Nkd1*) as well as the Wnt secretion regulator *Wntless* and the Wnt inhibitors *Notum* and *Dkk* (Additional file 8: Table S6, Additional file 9: Figure S4 and Fig. 3d). Another cluster included genes that were initially downregulated and then upregulated in later time points with some returning to the baseline by hour 72 (Fig. 5a2). This cluster contained Wnt6 and 10, Frizzled2, two Wnt co-receptor LRP adapter protein genes and two protocadherin fat4 like genes, which are associated with planar cell polarity (PCP) activity and growth control via the Hippo pathway [70, 71]. The third cluster contained genes that were upregulated at a later stage (Fig. 5a3), beginning at hour 24, and was thus termed “late upregulated”, with only three Wnt factors: *Wnts1*, *2* and *A*, which are closely related in a Wnt family cladistics analysis as reported by Hensel et al. [72] but are more distantly related according to Kusserow et al. [73]. This clustering of different pathway components indicates a pattern of co-regulation, elucidation of which may be important for deciphering the different stages of the oral vs. aboral patterning process in regeneration in the future studies.

Regarding the physal side, the majority of Wnt pathway genes showed lower and often only very little expression as compared to the oral side and thus we did not manage to cluster these genes’ expression patterns. A notable exception to the oral preponderance of the Wnt pathway members was the *Frizzled5* gene which was strongly upregulated at 8 h in the aboral part, while its oral expression was downregulated. *Nematostella Wnt5*, reported to function as a non-canonical PCP/convergent extension regulator in experiments conducted on *Xenopus* embryos [74], displayed a unique higher aboral as compared to oral expression pattern along the regeneration time course, except at 72 h when its oral levels were higher. Thus Wnt5 may signal via its cognate receptor Frizzled5 in the aboral side to allow elongation of the physa after the wound repair stage (see hypothetical model at Fig. 5b).

In the oral side the late *Wnt5* expression may likely induce evagination of tentacles as was reported in an elegant work on Hydra [75]. *Wnt5* is expressed in *Nematostella* at the base of tentacles [73], however the PCP function here may also be carried out by the non-canonical *Wnt11* [74], which is also expressed specifically in the tentacles at the juvenile polyp stage [76], and is an upregulated oral specific gene in our screen (Additional file 8: Table S6 and Additional file 9: Figure S4). It is not clear which Frizzled may mediate the presumptive Wnt/PCP program at the oral side but a possible candidate is *Frizzled2*.

Of interest, a Wnt/PCP pathway transmembrane co-receptor *Ror2*, exhibited a high aboral expression pattern similar to that of *Frizzled5*, which can indicate that this gene is also an evolutionarily conserved morphogenetic gene active in our speculative model of aboral PCP patterning at early regeneration (Fig. 5b). Regarding inhibitors of the pathway, besides the aforementioned *Notum*, we detected a *Dickkopf* (*DKK*) factor [76, 77] with an early oral expression and a steadily rising aboral expression reaching high levels at 72 h. We also found *APCDD1* [78, 79] (Table 1), a little known membrane-residing inhibitor reportedly studied only in humans until now, which is downregulated upon regeneration and whose expression increases only at the aboral side at the late stage. This expression mode may imply regulation at both sides of the canonical Wnt pathway at the earlier stages and a late establishment of the steady state Wnt oral to aboral gradient.

The dynamic and intricate expression mode of the Wnt factors that is usually higher in the oral regenerating regions but is often accompanied with a lower and shorter aboral activity may also imply that these genes play non-redundant roles in specifying different regions and structures along the oral-aboral axis in regeneration, partly recapitulating the embryonic/larval developmental patterning of this major axis in cnidarians [19, 22]. In Hydra *Wnt3* is the leading Wnt factor in head formation, budding, and head regeneration, and is expressed in the hypostome at the apex of the head [20, 22, 80]. In *Nematostella Wnt3* is expressed in the pharynx of the polyp [76], and we did not detect a response of the *Nematostella Wnt3* ortholog in our regeneration screen. Thus, the identity of the lead early Wnt factor in *Nematostella* regeneration remains to be determined. A possible candidate is *Wnt4*, which was upregulated very early on and had both the highest absolute reads and the largest fold induction level along the regeneration time course. This said, other early Wnt factors may induce the Wnt response and functional studies could resolve this issue in the future. Early onset of expression was detected for *Wntless*, *Frizzled1*, *Lef-1* (Additional file 9: Figure S4 and Fig. 3d) and many other Wnt pathway components, demonstrating that its signaling activity is turned on early in the process at both sides. We did not detect induction of β-catenin response but this does not preclude its activation at the protein level along with other members of the pathway, which may or may not be also induced transcriptionally.

In summary, the above reported observations allow us to propose a speculative model, in which the less explored aboral regeneration plan involves an early transient canonical Wnt pathway activation which may specify the aboral domain as such and eventually elicit cell proliferation (Fig. 5b). This stage may then be followed by the activation of the non-canonical (PCP) pathway either directly by the canonical pathway or indirectly by aboral specific TFs. Activation of the Wnt-PCP pathway may induce elongation of the aboral part and may participate in the inhibition of the canonical pathway at later times together with the canonical inhibitors such as DKK (Fig. 5b). Thus an intricate interaction between Wnt pathways may lie at the basis of the regeneration process and other cues may also be involved such as ciliogenesis as described ahead. Future experiments in which the canonical and PCP Wnt pathways will be manipulated using pharmaceutical agents or genetic tools will allow establishing our proposed model.

#### Additional developmental signaling pathways

Although the GO analysis did not point to other enriched pathways showing differential expression, we manually searched for highly differentially expressed genes belonging to other families of signaling factors known to be involved in development and regeneration, such as the BMP, Hedgehog, FGF and Notch pathways (see Methods). Indeed we have located much fewer genes with a low CCC score (<0.6) for each of these pathways as compared to the Wnt pathway (Additional file 10: Table S7), explaining why they did not show up in the pathway GO analysis. Nevertheless, there were several individual members of these families that were considerably differentially expressed (Additional file 10: Table S7). These included the following: 1. two genes coding for ligands of the FGF pathway. 2. *Hedgehog* and its receptors *patched domain-containing protein 3-like* and *Smoothened* and the zinc finger *Zic1-like*. 3. the Notch pathway *Jagged1* and *Delta-like* ligands, a HLH domain *Hes4-like* TF gene and a *Hey*, a Notch transcriptional repressor gene. Thus, these pathways may also contribute to the polarized regeneration process even though their participation seems to be less extensive than that of the Wnt pathway, which displays many DE components at all levels of the signaling cascade. Whether there are specific roles for the different pathways in regeneration and their possible interaction with the Wnt pathway are interesting avenues for future research.

With regard to the BMP pathway, most of its ligands and receptors were non-responsive or only weakly expressed, except for BMP2/4, which was downregulated during the course of regeneration (Additional file 10: Table S7). Of note was the *Smad4* effector gene which did show induction of expression, an observation that is in line with recent work on Hydra head regeneration [68]. Interestingly, the *Tolloid* gene (*Nve-Tld* [81]), which encodes for an Astacin type metalloproteinase that has multiple substrates including the BMP2/4 antagonist Chordin [82], showed a shift in expression from high oral to aboral with time. However, when we examined BMP antagonist like genes, we found that while *Chordin* and *Noggin* orthologs had very low time course responses, *kielin/chordin-like (KCP)* [83], a gene whose product enhances BMP signaling, displayed high aboral expression. These findings may suggest that the BMP pathway functions differently in regeneration as compared to its activity during development in *Nematostella*.

#### Chitin-related genes

Surprisingly the group of genes that was most highly enriched in our analysis of differential expression was the chitin associated genes (Fig. 4 and Additional file 11: Figure S5). This group contained mostly *chitinase* genes, as well as *von willebrand factor d* and *egf domain-containing* genes that are also predicted to be secreted and to harbor a chitin binding domain found in animal chitinases. These enzymes have been reported to be expressed in the endoderm of the body column of three Hydra species [84] and were suggested to function in prey digestion. The first cnidarian chitinase that was cloned and studied in the hydroid *Hydractinia*, was rather found to be highly expressed in the ectodermal layers of the stolons of the colonies and of the basal (aboral) part of the polyps, and is proposed to act in immunological protection of the latter from chitinous pathogens [85]. However, some of the chitin associated genes that we located in the screen were chitin synthases and chitin deacetylases, which may strongly hint that *Nematostella* contains chitin, as do other anthozoans such as corals and a shell producing sea anemone [86].

Interestingly, all of the above chitin associated genes show a similar time course expression pattern upon regeneration; at time 0 the oral facing proximal region has a higher level of expression as compared to the aboral facing region, which is typical of genes exhibiting a sharp gradient in the mid-gastric zone. At later time points both of the oral and aboral expression levels decline, with some of the genes displaying late upregulation of aboral expression, which likely restores the initial aboral high/oral low gradient (Additional file 11: Figure S5). Thus, while the function of these chitin-related genes is yet unexplored, our study indicates that the activity of this gene assembly is quickly downregulated upon regeneration and is restored towards its later stages in order to re-establish the aboral specific steady-state function.

#### Tubulin and cilia associated genes

**Fig. 6:**
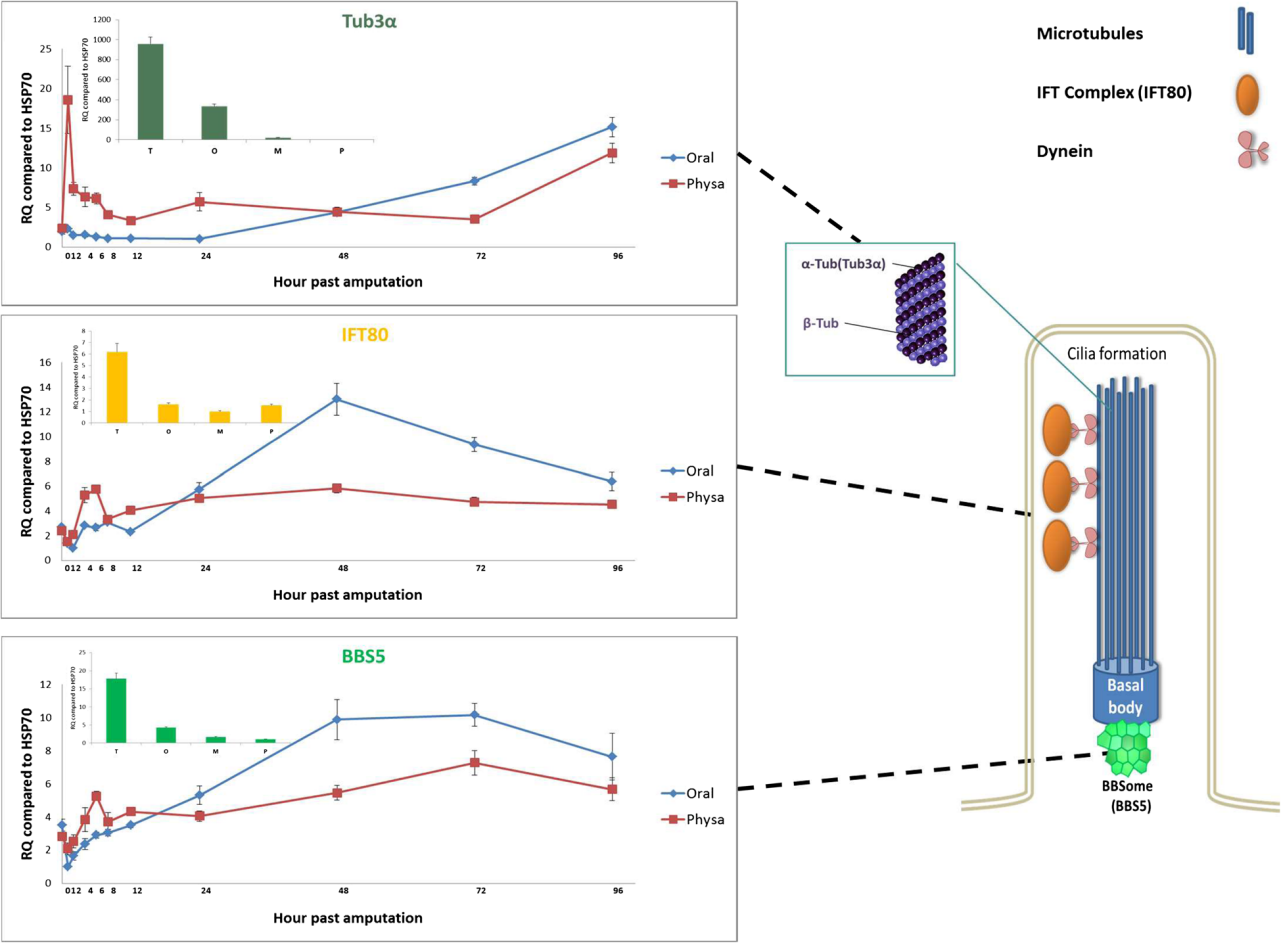
Tubulin and cilia-associated genes in regeneration. A schematic depicting the possible involvement of tubulin and cilia enriched gene products in aboral regeneration. The axoneme of the cilia together with the basal body and its associated BBSome complex (see text) are illustrated. The graphs of representative early aboral enriched genes that are known to reside in cilia are shown: one of the two *Tubulin3α* whose products are part of the microtubules; *IFT80*, which is part of the dynein-driven IFT protein complex; and *BBS5*, which is a member of the BBSome coat complex that is linked to the basal body of the cilia. The inset in the *Tubulin3α* panel depicts the expression level in different body parts of intact polyps: T, tentacles; O, oral ring and pharynx; M, mesenteries and P, physa. IFT, intra-flagellar transport; BBSome, Bardet-Biedl Syndrome protein complex

An additional GO group of interest was that of microtubule cytoskeleton, which stood out in the side-dissimilarity of the expression patterns of its members, although it was not one of the GO groups that were found to be enriched in the screen (Fig. 4). Further inspection of this group pointed to a number of genes, among them two *tubulin-alpha3* genes, which are highly expressed in the aboral regenerating region at the early stages (Fig. 6 and Additional file 12: Table S8). This was surprising as these genes as well as many other microtubule-associated genes, are more highly expressed in the oral part in intact animals, compared to the aboral. Most interestingly, there was also high aboral expression of some cilia-related genes during regeneration (Fig. 6). These include IFTs (intraflagellar transport proteins), that are important for cilia formation and function, and several tubulin polyglutamylases, which modify tubulins into ciliary-enriched forms [87]. Another early aboral expressing gene we identified was the *Bardet-Biedl syndrome protein 5 homolog* (*BBS5*) gene, which is part of the basal body linked complex of proteins (BBSome) [88] that are mutated in genetic diseases linked to ciliary function, termed ciliopathies. All of this evidence suggests that an early ciliogenesis reaction occurs in the aboral regenerating region of *Nematostella*, which may imply that cilia are involved in aboral regeneration. This can be attributed to several possible mechanisms such as the directed flow of secreted factors in the gastrovascular lumen or cilia-based signaling.

Regarding the latter, it was established that components of the hedgehog pathway reside in cilia, and that cilia are essential for the function of this pathway in mammalian development [89]. We did not find, however, any conclusive concerted aboral specific induction of the major hedgehog pathway genes upon regeneration (Additional file 10: Table S7) but this cannot negate the possibility that there is an aboral enrichment at the protein level. Other pathways have been proposed to affect, and be affected by, cilia formation. Of these it is worthy to note that disruption in several ciliary genes elicited the canonical Wnt pathway in some developmental scenarios [90, 91]. Thus, ciliogenesis may be expected to have an effect on the inhibition of Wnt signaling. Accordingly, we can speculate that if indeed our tubulin and cilia-associated expression data indicates that cilia formation takes place in the aboral regenerating animals and this may explain the early down-regulation of the canonical Wnt response which we observed as part of the aboral reaction (Fig. 5b).

### An evolutionary comparison between *Nematostella* and Planarians

**Fig. 7:**
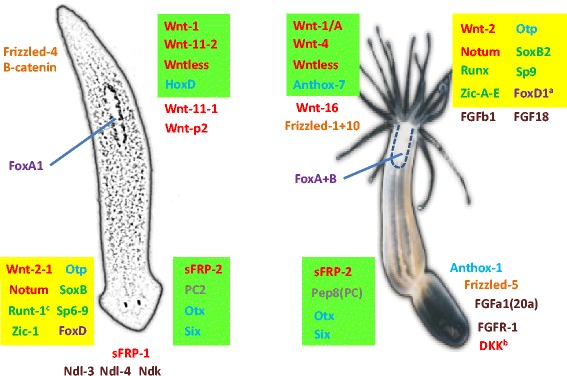
Comparison of “head” vs. “tail” gene expression in the regeneration of *Nematostella* and Planaria. A cartoon of the two animals is presented with the oral (head) part of *Nematostella* aligned with the posterior (tail) part of Planaria according to the comparison in which these ends express an assembly of *Wnts* and other universal bilaterian posterior markers, while the aboral part resembles the Planarian anterior (head) part in the expression of universal anterior markers such as *Six3/6* and *Wnt* inhibitors like *sFRPs* [51]. Some of the markers specific for the main axis ends are depicted with *Wnts* and *Wnt* pathway genes in *red* and *orange*, *FGF* pathway members in *brown*, homeobox factors in *light blue*, forkhead (Fox) factors in *purple*, and other TFs in *green*. An anterior Planaria regeneration marker and protease, proprotein convertase 2 (PC2) and its ortholog in *Nematostella*, the late aboral *Pep8* gene are marked in *grey*. Highlighted in *light green* are *bona fide* orthologs that are expressed as expected according to this comparison, while *yellow* marks polarized genes that are expressed in an ‘inverted’ position, which can indicate evolutionary change in the regeneration program. ^a^The *FoxD1* gene is induced at the oral regenerating region in *Nematostella* however it is aboral in embryonic/larval development and becomes orally expressed upon metamorphosis and tentacle budding [95]. ^b^ *Dickkopf* (*DKK*) is expressed higher orally at 8 h after the induction of regeneration and higher in the aboral part later than 24 h. ^c^ *Runt-1* is induced in anterior sites upon eye and neural brain regeneration in Planaria but is also expressed in posterior wound sites [35, 37] and https://radiant.wi.mit.edu/app/

Finally, we compared the expression patterns of the key genes we identified in our screening to those reported in the well-studied regeneration plan of the Planaria, where the head vs. tail regeneration program has been studied in depth [24, 27, 28, 38, 92]. To this end, we identified the *Nematostella* orthologs of the known Planaria anterior and posterior marker genes which are also induced during regeneration in a polarized fashion. When no bona fide orthologs could be found we added the closest homologous genes that also show polarized expression to complete the picture (Additional file 13: Table S9 and Fig. 7). Many of our oral-induced genes corresponded to the tail/posterior genes/markers of the Planaria and inversely, the aboral genes matched those of the head/anterior Planarian genes (Fig. 7). This relationship between the cnidarian oral/aboral axis and the anterior/posterior axis of bilaterians has been reported recently [51], where the aboral part of *Nematostella* shows a molecular resemblance to the anterior part of bilaterians, and inversely, the oral region has similarity to the posterior bilaterian side. Thus, the aforementioned master aboral territory gene *Six3/6* [51] and the *OtxC* gene that are highly expressed in aboral regeneration (Fig. 3d) have orthologs in Planaria that are anteriorly expressed (Fig. 7). We also detected a higher aboral expression of *FGF1a* [93] (*Fgf20* in [57]) and *FGF-R1- like* with a corresponding head expression of the FGF-receptor like *ndk*, *ndl-3* and *ndl-4* Planaria genes. Interestingly, we also found several oral enriched *Nematostella* FGF factors induced in regeneration (Fig. 7), however, the identity of their receptors as well as of the aboral FGF factors and their effect in each side awaits further studies. Interestingly, we found that the forkhead factors *FoxA2* and *FoxB* in *Nematostella,* which were reported to be expressed in the pharynx during larval development and in young adults [94, 95], have a late high oral expression pattern. Accordingly, they most likely correspond to their Planarian counterpart *FoxA*, which was shown to be a pharynx marker [96] and a factor that is essential for the formation of this organ in Planaria and perhaps in all eumetazoans that contain pharynx/foregut [97].

Notably, several *Wnt* genes are expressed in the posterior side as well as in the tail forming side of wounds in the Planaria and in the corresponding oral regenerating side of *Nematostella*, while the Wnt inhibitor sFRP is expressed in head facing wounds of Planaria and in the aboral regenerating side of *Nematostella* (Fig. 7). Although we cannot determine at this stage that the orthologs have a similar role in the regeneration process in these two phylogenetically remote animals, some similarities can be observed. For example, the involvement of several *Wnts* exhibiting early and late induction times such as *Wnt4*, which is initially expressed at both the regenerating sides (Fig. 3), can be likened to the key Planarian *Wnt1* (*WntP-1*) that is initially expressed at both wound faces before becoming enriched at the posterior side [28], and induces later expressed *Wnt* genes. Additional examples are *Frizzled1* and *10* which are orally expressed in *Nematostella* and may correspond to *frizzled4* which is posterior in Planaria. We did not observe any polarized expression of the major effector β-catenin in *Nematostella*, while in Planaria it is a key posterior gene in regeneration [24]. Whether these homologous genes function similarly should be established in future perturbation experiments where the phenotypic and molecular effects can be compared.

However, some orthologs of the two species are inversely expressed with respect to the above rule. This could be seen for a group of side specific transcription factors including *orthopedia* (*Otp)*, *Zic*, *SoxB* and *FoxD* which we found to be oral in *Nematostella* regeneration but are anterior in Planaria. Besides these factors, *Wnt2* in Planaria is unusual as it is more anteriorly expressed in relation to all its other paralogs that are posterior oriented [28]. Most interestingly, the Wnt inhibitor *Notum* is orally expressed in *Nematostella*, while it is anterior in the Planaria [29]. This inversion of *Notum* expression in Planaria is of special interest as it was very recently found to represent the most early asymmetrically expressed gene in the Planaria [35], and thus orchestrates the subsequent polarization of Wnt factors [29], which is crucial for establishing the polarity of the A-P axis. *Notum* was found to be posteriorly induced in regeneration of the acoel worm *Hofstenia* [98], which resembles the *Nematostella* expression pattern with regard to the *Wnt* factors expression, and thus the Planarian regeneration scenario may be derived as compared to the basal state. It is interesting to note that we did not detect genes that are expressed in the aboral part of *Nematostella* which have their orthologs expressed in the posterior domain of the flat worm. A simple explanation is that besides the *Wnt* factors not many genes were defined in this part in Planaria, while the anterior region is more complex in term of structures and has also been more explored. However this finding could also suggest that the oral part of *Nematostella* has indeed similarities to both the anterior and the posterior parts of the Planaria and may attest to grand maneuvers which occurred in axis polarity along evolution.

Such changes in the polarity of gene expression along the major axis may reflect macro-evolutionary changes owing to differences in the body plan of bilaterians compared to that of radial-like symmetry in cnidarians, or in the regeneration mechanisms employed by these two evolutionary distant animals that also exhibit very different life styles. Understanding the basis for these evolutionary changes in the regeneration plan will also benefit from elucidating the gene networks involved and insight may be gained regarding the nature of changes that sculpt different body plans and the evolutionary process that shaped them. Eventually, the study of the mechanisms underlying regeneration in multiple animal models may be a most insightful approach to elucidate the molecular basis of this fascinating developmental process and may prove beneficial for future innovative injury treatments in man.

## Conclusions

The re-formation of polarity upon the course of regeneration is a long time biological mystery but in general terms it is a specialized case of the formation of the polarized body of animals that occurs upon development. While the overall response in the two sides seems to be very similar with many genes being downregulated, there were nevertheless many genes which were expressed in a side-specific manner. Three main groups of genes were differentially expressed between the sides, namely proteases, transcription factors and Wnt pathway members, with the last especially demonstrating a complex dynamic expression pattern, suggesting an interplay between the canonical and non-canonical pathways at both sides. Among the other polarized groups, the microtubule and cilia associated genes stood out as novel potential players in the control of polarity induction. From an evolution wide comparison of the *Nematostella* regeneration plan to Planaria, we can discern a similarity of a conserved developmental pathway and other key genes along the presumed corresponding sides of the main axes of these animals, while multiple genes also show an inverse pattern of expression. Thus, like development in general, the whole body regeneration plan seems to harbor a common general trend with variations evolved to accommodate the specific body shape and structure of different animal groups.

## Methods

### *Nematostella vectensis* culture and regeneration

*Nematostella* polyps were raised and kept in 30 % artificial sea water (ASW) at 18° and fed freshly hatched *Artemia* three times a week. Adult 4 months old polyps of 1.5–2 cm length were used in the regeneration experiments, prior to which their water was changed and they were not fed for 3 days. Animals were relaxed in 7 % MgCl_2_ in 30 % ASW for 10 min prior to bisection halfway between the oral and aboral ends. For the hour 0 time point, a 1 mm wide section was collected from the area of the cut immediately following bisection. Regenerating *Nematostella* were kept in 30 % ASW at 18° and the regenerating ends were sampled at 8, 24 and 72 h following the bisection. At those specific time points a 1 mm wide section was taken from the areas undergoing regeneration and collected in Tri-reagent (T9424 Sigma), homogenized and frozen at −20° until further use. The animals assayed were cut twice: once upon the initial bisection and once again at the time of sample collection. 20 animals were used per sample with two biological replicates per time point, for a total of 16 samples (4 time points x 2 regeneration areas x 2 replicates). For quantitative PCR experiments the same procedure was performed but *Nematostella* were sampled at 0, 1, 2, 4, 6, 8, 12, 24, 72 and 96 h post amputation. Pictures of the area of regeneration (Fig. 1) were taken at times corresponding to those sampled for sequencing and used a Nikon DS-Fi1 with DS-U2 controller (resolution 2560X1920).

According to the ethics instructions of the Hebrew University of Jerusalem most invertebrates including cnidarians such as *Nematostella* are exempt from the ethics committee inspection.

### RNA isolation and sequencing library preparation

RNA from the tissue samples was processed in Tri-reagent as described [99]. Libraries were prepared from RNA samples using Illumina TruSeq RNA Sample Preparation Kit v2 (Illumina # RS-122-2001) according to the manufacturers’ instructions. This protocol utilizes poly-A selection of mRNA from total RNA. Library concentrations were quantified with Agilent 2200 Tapestation, diluted to 10 nM and pooled with 8 samples in each pool. The samples were sequenced using 50-bp single-end reads on two Illumina HiSeq2000 lane and TruSeq v3 flow chambers at the The Technion Genome Center, Haifa, Israel.

### Processing and RNA-Seq data analysis of Illumina sequencing reads

*Nematostella vectensis* reference genome was downloaded from Ensembl. Gene product annotations were retrieved from UniProt. However, proteins designated as ‘predicted protein’ were annotated using Blast2GO through blastp vs. refseq (considering up to top 20 hits, *e*-value cut-off 10^−3^).

The 50-bp reads were quality assessed using FASTQC and subsequently aligned to the *N. vectensis* genome using the STAR aligner [100]. Only those reads uniquely aligned to the reference genome were subsequently used for analyses. Raw read counts per gene were counted using HTSeq-count. Subsequent normalization and differential expression analyses were carried out using the DESeq package [59, 101]. As we used duplicates in this experiment, we decided not to perform pairwise comparisons among pairs of time points, but instead perform a test which considers the entire time course in both regeneration-sides. Using DESeq, we applied a *multi-factorial design*, with the time and regeneration-side as the biological factors influencing gene expression. For each gene, we checked whether the time in general had a significant effect on its expression, while accounting for the effect of the side of regeneration. In short, for each gene, two generalized linear models (GLMs) were fitted using the fitNbinomGLMs function: a full model regressed the genes’ expression on both the regeneration-side and the time effect; a reduced model regressed the gene expression only on the regeneration-side effect. The nbinomGLMTest was then used to compare the two models in order to infer whether the additional specification of the time (on top of the regeneration-side) improved the fit and hence, whether the time had a significant effect on gene expression. This analysis resulted in a *p*-value per gene, which was further adjusted for multiple testing using the BH method. Out of >23,000 expressed genes, 4205 genes had time effect adjusted *p*-value < 0.05. These genes were termed “differentially expressed” (DE) genes. These genes were then further analyzed throughout the rest of this work.

### Clustering analyses

Hierarchical clustering of DE genes was performed in Partek® Genomics Suite® using Pearson’s dissimilarity and complete linkage. Prior to hierarchical clustering, the normalized counts were log2 transformed (after zeros were converted to 1) and standardized (z-scored) such that gene-wise means were shifted to zero and the standard deviation was scaled to 1. Partition clustering of DE genes was performed with STEM [102] using the STEM clustering method and a maximum of 20 model profiles.

To determine up- or down-regulation among certain time points in a given side, a difference of at least 1.6 fold between the time points was required, considering average of normalized counts per time point. We chose to use a rather permissive fold change cutoff since we were looking at the changes across a few time points together so that even a slightly lower but consistently changed expression was significant. A gene was considered to be always down or upregulated during regeneration if the expression level of all time points were at least 1.6 fold lower or higher than hour 0 respectively. DE genes that didn’t fall into these categories were counted as "other". For comparison between consecutive time points a similar process of 1.6 fold change was used, albeit between each time point and its previous one, to determine the overall change in transcriptome expression levels. For *Wnt* family genes, hierarchal clustering was performed using the heatmap.2 function in R, and the tree was separated to discrete clusters using the cuttree function.

### Concordance calculation among regeneration sides followed by Gene Ontology (GO)

To assess the concordance or discordance of the time-dependent expression pattern between the oral and aboral sides of regeneration we calculated Lin’s concordance correlation coefficient (CCC) [103] for the 4 data points of oral and physal regeneration. This calculation was performed on the 4205 DE genes defined above. CCC ranges from 1 to −1 with different consensuses as to what constitutes a poor concordance. Based on observations of the data we found that genes with a CCC of 0.6 or less showed little to no similarity between sides and 0.6 was thus chosen as a cutoff for capturing genes with different expression patterns between regenerating sides. Genes with CCC < 0.6 were defined as having low concordance, and were subsequently analyzed for enriched GO terms using the DAVID server [104–106]. A full list of enriched GO terms can be found in Additional file 4: Table S3. Many enriched GO terms contained similar genes and only one from each grouping was shown in Fig. 4.

To define *Nematostella* genes from several pathways of interest, gene lists were taken from KEGG (Kyoto Encyclopedia of Genes and Genomes). Since the KEGG *Nematostella* pathway annotation is not complete, pathway genes not available in the *Nematostella* annotation of KEGG were supplemented by using BLAST to find genes corresponding to prominent pathway orthologs.

### Quantitative real-time PCR (qRT-PCR) analysis

To confirm gene expression levels detected by RNA-Seq, quantitative real-time PCR was performed using the Applied Biosystems StepOnePlus™ Real-Time PCR System using gene-specific primers (Additional file 14: Table S10). RNA was extracted using Tri-reagent [99] and RNA integrity was verified using agarose gel electrophoresis. RNA concentration and purity was measured using a NanoDrop spectrophotometer. cDNA was synthesized using High-Capacity cDNA Reverse Transcription Kit (Applied Biosystems). cDNA samples were run using Fast SYBR® Green Master Mix (Catalog Number 4385612/4). Relative quantity calculations were performed with the ΔΔCt method, normalized against the gene Nv-Hsp70 as described [56]. The error bars represent SD of three technical repeats and the graphs are representative of at least 2 biological repeats performed on different batches of animals. In general about 75 % of the genes analyzed by qRT-PCR showed overall similarity in their expression patterns to the results of the RNA-Seq.

## Additional files

Additional file 1: Table S1. The differentially expressed (DE) genes of the regeneration screen. The list contains the gene id, followed by Uniprot or Blast2GO annotation and FDR adjusted *p*-value of the time effect. This is followed by the average reads for each time point, DESeq normalized read count per each duplicate time point and raw read count per each duplicate time point. The annotation (−−) appears when Blast2GO did not retrieve any blast hit in the RefSeq protein database. (XLSX 2933 kb) Additional file 2: Figure S1. Clusters of DE oral and physal genes. List of all 20 clusters of oral and aboral expression produced by STEM (see Methods). Clusters are ordered by number of genes in each cluster. Numbers in the top left corner represent the cluster number. Numbers in the bottom left corner represent the number of genes in each cluster. A list of DE genes and the cluster they belong to can be found in Additional file 3. (PDF 213 kb) Additional file 3: Table S2. DE genes and their CCC. List of all DE genes and their time effect FDR adjusted *p*-value and CCC. In addition, the side of higher expression in most of the time points and the side and hour of the highest level of change in expression per gene are given. The cluster the gene belongs to (from Fig. 2c and Additional file 2: Figure S1) is also indicated, as is whether the gene is consistently expressed throughout regeneration (Fig. 3a) (XLSX 524 kb) Additional file 4: Table S3. Enriched GO terms in low CCC genes. GO terms containing similar genes are colored similarly. Additional tabs contain a list of GO annotated low CCC genes belonging to the GO terms mentioned in Fig. 4 and a list of all the corresponding GO terms associated with each gene. (XLSX 575 kb) Additional file 5: Table S4. Metalloproteinase (MP) groups and inhibitors. A list of the major DE MPs identified in the screen with *ADAMTS*, *Astacins*, other *MPs* and their inhibitors. (XLSX 676 kb) Additional file 6: Figure S3. Time course graphs of *metalloproteinase* (*MP*) group genes and inhibitors. The FDR-adjusted *p*-values for the time effect (DESeq GLMs) for all genes are given. (XLSX 23 kb) Additional file 7: Table S5. Homeobox and other TF groups that are highly DE. (XLSX 790 kb) Additional file 8: Table S6. Wnt family genes clusters. A list of the cluster groups of the Wnt family genes and a list of the genes in each cluster shown in Fig. 5a. (XLSX 11 kb) Additional file 9: Figure S4. Time course graphs of the major DE Wnt factors and Wnt pathway members. (XLSX 81 kb) Additional file 10: Table S7. DE genes of different developmental pathways. A list of DE genes of major the developmental pathways that were manually detected (see Methods) and have a CCC < 0.6. The time-course graphs of prominent pathway genes are also shown. (XLSX 625 kb) Additional file 11: Figure S5. Expression of chitin upon regeneration. A. Expression patterns of representative chitin genes in our screen showing the difference in their oral and aboral expression levels at the hour 0 time point. B. A scheme illustrating the possible gradient patterns in intact *Nematostella* as suggested by qPCR on the different parts. These gradients can explain the early difference in expression level and the later return to the original polarized expression levels. (PDF 1722 kb) Additional file 12: Table S8. Time course graphs of the major DE tubulin and cilia associated genes. (XLSX 40 kb) Additional file 13: Table S9. Comparison of polarized gene expression in regeneration between *Nematostella* and Planaria. The anterior and posterior enriched genes of Planaria are listed with their oral or aboral *Nematostella* orthologs and homologs when present. The color coding is the same as in Fig. 7. (XLSX 17 kb) Additional file 14: Table S10. A list of the primers that were used for qRT-PCR experiments. (XLSX 11 kb)

## Abbreviations

ADAMT: A disintegrin and metalloproteinase with thrombospondin motifs
AP: Anterior-posterior
ASW: Artificial salt water
BH: Benjamini & Hochberg
CCC: Concordance correlation coefficieant
COMP: Cartilage oligomeric matrix protein
DE: Differentially expressed
DEG: Diferentially expressed gene
DKK: Dickkopf
ECM: Extracellular matrix
FPKM: Fragments Per Kilobase of transcript per Million fragments mapped
GLM: Generalized linear models
GO: Gene ontology
GRN: Gene regulatory network
IFT: Intraflagellar transport protein
MMP: Matrix metalloproteinase
MP: Metalloproteinase
PCP: Planar cell polarity
qPCR: quantitative PCR
SD: Standard deviation
STEM: Short time-series expression miner
TF: Transcription factor
TIMP: Tissue inhibitors of metalloproteinase
