# Supplementary figures and images for: A transcriptional time-course analysis of oral vs. aboral whole-body regeneration in the Sea anemone Nematostella vectensis
Source: BMC Genomics. 2016 Sep 7;17(1):718. doi: 10.1186/s12864-016-3027-1 (PMC5015328; doi:10.1186/s12864-016-3027-1)

# DE Oral clusters

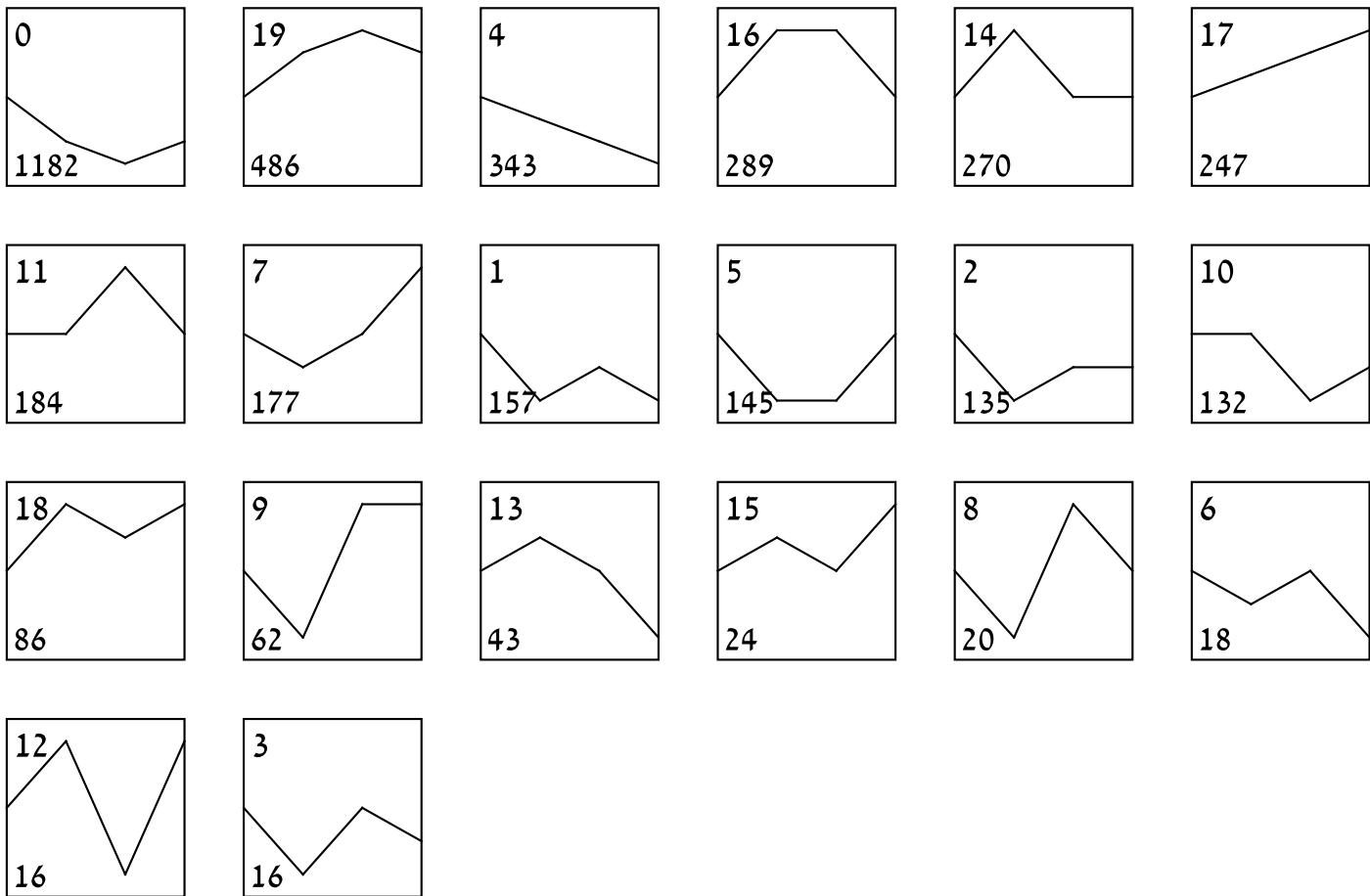

# DE Physa clusters

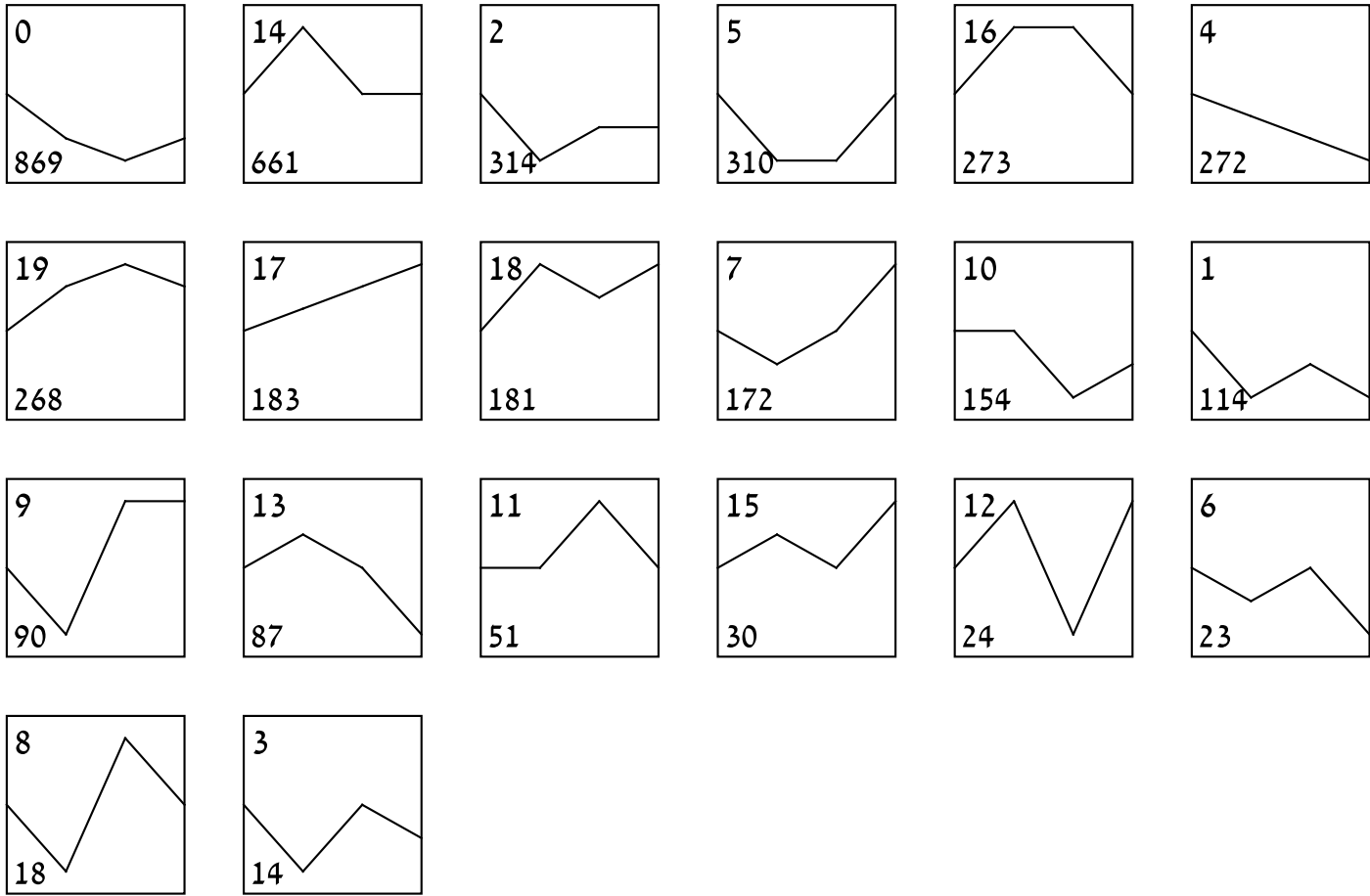

Profiles ordered based on the number of genes assigned

Supplement: Additional file 2: Figure S1. — Clusters of DE oral and physal genes. List of all 20 clusters of oral and aboral expression produced by STEM (see Methods). Clusters are ordered by number of genes in each cluster. Numbers in the top left corner represent the cluster number. Numbers in the bottom left corner represent the number of genes in each cluster. A list of DE genes and the cluster they belong to can be found in Additional file 3. (PDF 213 kb) [file 12864_2016_3027_MOESM2_ESM.pdf]

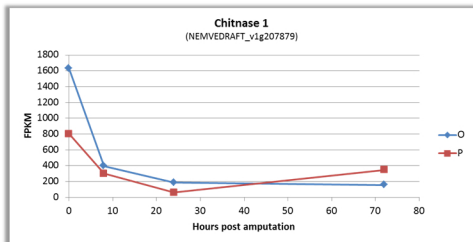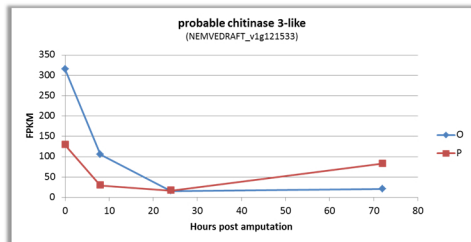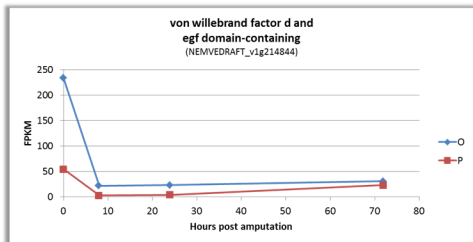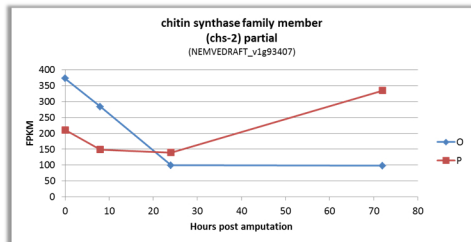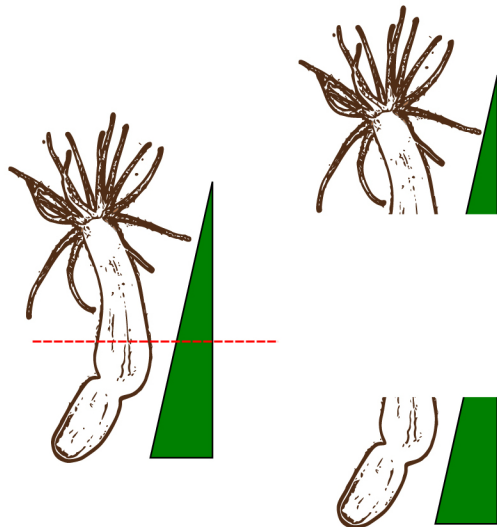

Supplement: Additional file 11: Figure S5. — Expression of chitin upon regeneration. A. Expression patterns of representative chitin genes in our screen showing the difference in their oral and aboral expression levels at the hour 0 time point. B. A scheme illustrating the possible gradient patterns in intact Nematostella as suggested by qPCR on the different parts. These gradients can explain the early difference in expression level and the later return to the original polarized expression levels. (PDF 1722 kb) [file 12864_2016_3027_MOESM11_ESM.pdf]
